# Supplementary material for: Organic Molecule Assisted Growth of Perovskite Films Consisting of Square Grains by Surface-Confined Process
Source: Nanomaterials (Basel). 2021 Feb 12;11(2):473. doi: 10.3390/nano11020473 (PMC7918920; doi:10.3390/nano11020473)
Supplement: Supplementary file 1 [file nanomaterials-11-00473-s001.pdf]

nanomaterials

MDPI

Supplementary Materials

Organic Molecule Assisted Growth of Perovskite Films Consisting of Square Grains by Surface-Confined Process

Shao Xin Yan [1], Chang Bao Han [1,*], Jianhua Huang [2], Yichuan Chen [1], Xiaobo Zhang [1], Xiaoqing Chen [1], Yongzhe Zhang [1,*] and Hui Yan [1]

[1] Faculty of Materials and Manufacturing, Beijing University of Technology, Beijing 100124, China; yansx@emails.bjut.edu.cn (S.X.Y.); lezhi2005@163.com (Y.C.); ZhangXB@emails.bjut.edu.cn (X.Z.); chenxiaoqing@bjut.edu.cn (X.C.); hyan@bjut.edu.cn (H.Y.)

[2] College of Petrochemical Engineering, Hunan Petrochemical Vocational Technology College, Hunan 414012, China; huangjhyy@163.com

* Correspondence: cbhan@bjut.edu.cn (C.B.H.); yzzhang@bjut.edu.cn (Y.Z.); Tel.: +86-187-0151-6966 (C.B.H.)

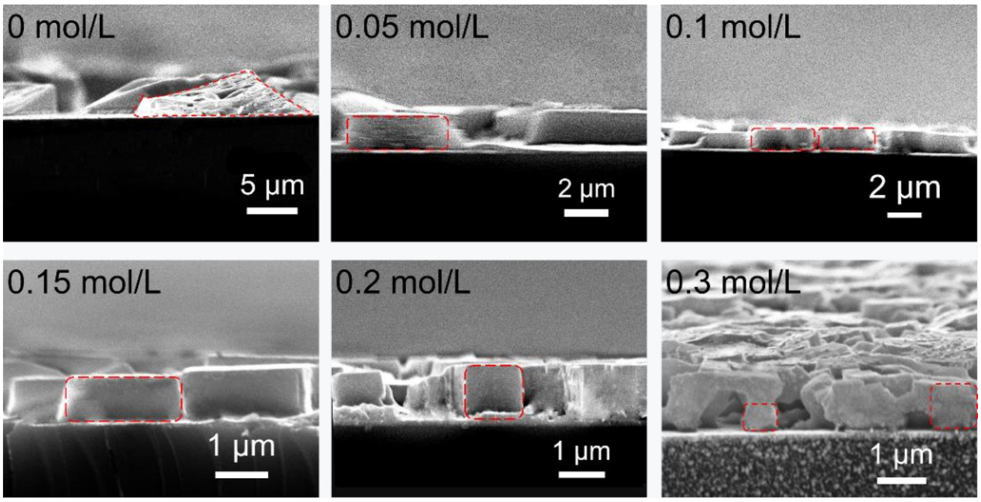

Figure S1. Cross-sectional view SEM images of perovskite films with various PEAI concentrations.

After the addition of PEAI, perovskite grains grew into squares under the effect of surface restrained. With the concentration of PEAI increases, the aspect ratio of grains gradually decreases. And the grains will be stacked due to the excessive nucleation amount when the concentration of PEAI is too much.

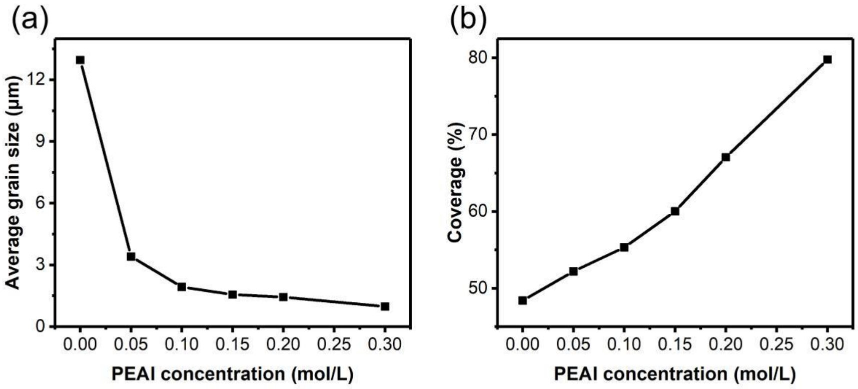

Figure S2. Average grain size (**a**) and coverage (**b**) of perovskite with different PEAI concentrations.

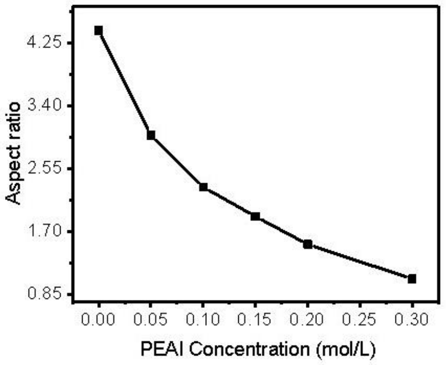

Figure S3. Correlation of grain aspect ratio and PEAI concentration.

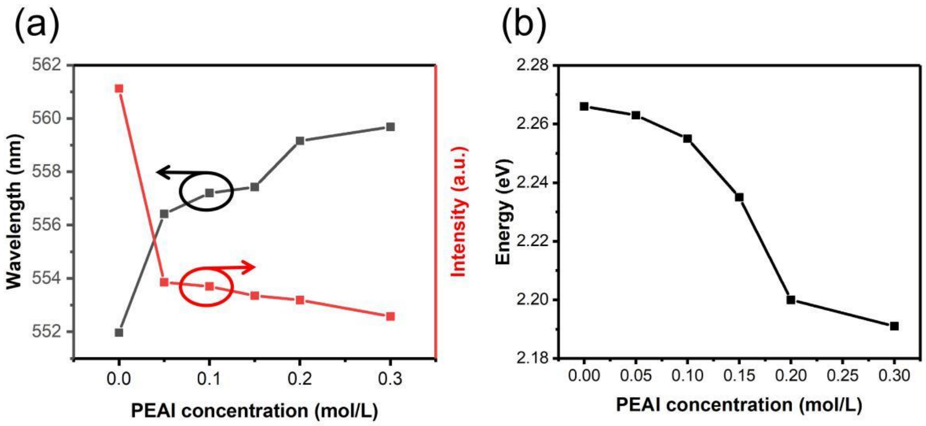

Figure S4. (**a**) The wavelength and intensity of the $MAPbBr_3$ emission peak at different PEAI concentrations. (**b**) Perovskite bandgap energy at different PEAI concentrations.

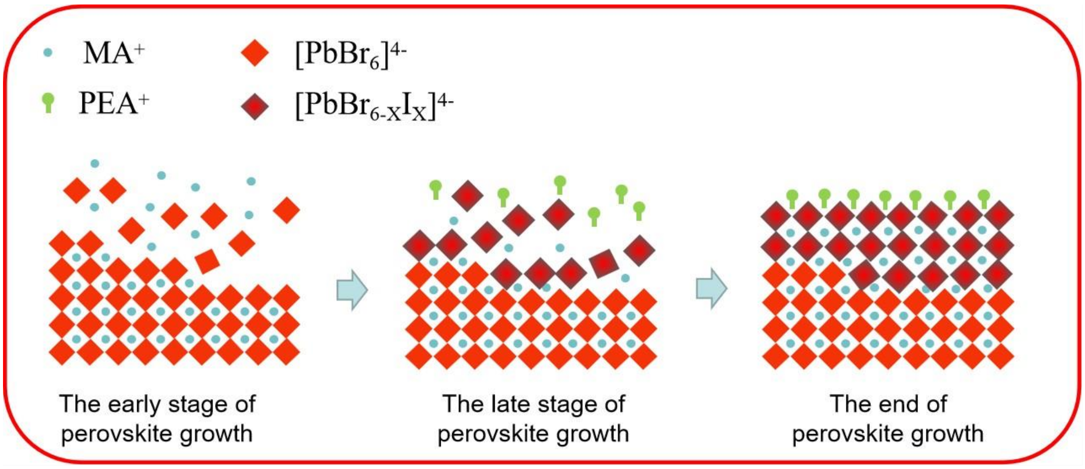

Figure S5. Schematic diagram of perovskite growth in precursor solution containing PEAI at different stages.

During the process of nucleation and early growth of perovskite, since the concentration of Br is much higher than I in the solution and Pb-Br bond is stronger than Pb-I bond [1–3], $[PbBr_6]^{4-}$ will be formed in solution rather than $[PbBr_{6-x}I_x]^{4-}$. Therefore, $MAPbBr_3$ perovskite first nucleates and grows in solution. In the late stage of perovskite growth, $[PbBr_{6-x}I_x]^{4-}$ begins to form in solution due to the decrease of Br concentration. Thus, $MAPbBr_{3-x}I_x$ epitaxy grows on $MAPbBr_3$.

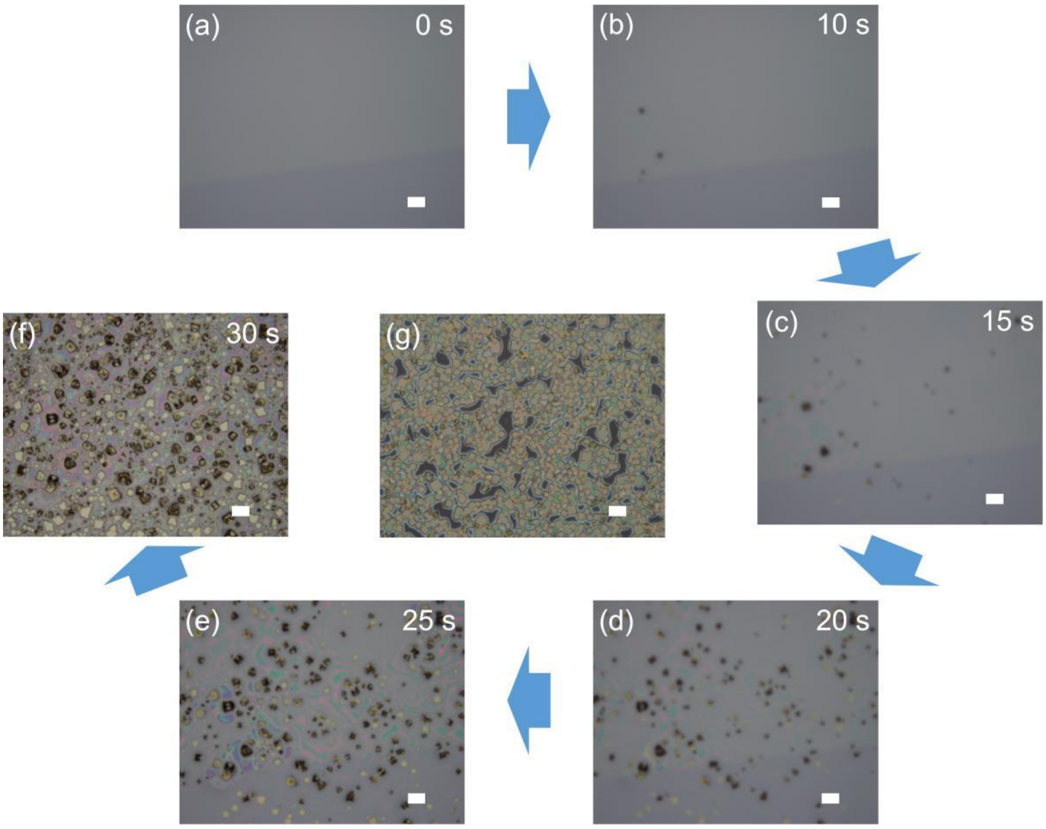

Figure S6. (**a**–**f**) Microscopic image of perovskite films with different standing times (The samples are only spin-coated for 5 s). (**g**) Microscopic image of the perovskite film prepared by spin-coated for 60 s. The scale bar is 50 μm.

Table S1. Grain size of the perovskite at various PEAI concentrations.

	**Min (μm)**	**Main (μm)**	**Max (μm)**	**Average (μm)**	**Range (μm)**
0 mol/L	7.54	10.45	29.65	12.96	22.15
0.05 mol/L	1.50	3.25	5.18	3.41	3.68
0.10 mol/L	1.13	2.05	3.06	1.93	1.93
0.15 mol/L	0.87	1.65	2.63	1.56	1.76
0.20 mol/L	0.77	1.35	2.41	1.44	1.64
0.30 mol/L	0.41	0.89	1.77	0.98	1.36

References:

1. Knight, A.J.; Herz, L.M. Preventing phase segregation in mixed-halide perovskites: a perspective. *Energy Environ. Sci.* **2020**, *13*, 2024-2046.
2. Benavides-Garcia, M.; Balasubramanian, K. Bond energies, ionization potentials, and the singlet-triplet energy separations of $SnCl_2$, $SnBr_2$, SnI_2, $PbCl_2$, $PbBr_2$, PbI_2, and their positive ions. *J. Chem. Phys.* **1994**, *100*, 2821-2830.
3. Yoon, S.J.; Stamplecoskie, K.G.; Kamat, P.V. How lead halide complex chemistry dictates the composition of mixed halide perovskites. *J. Phys. Chem. Lett.* **2016**, *7*, 1368-1373.
